# Mortality of Doctors

**Published:** 1878-12

**Authors:** 


The Mortality of Doctors.—The question is often asked, are we longer or shorter-lived than other classes of the community? The answer is difficult. If we take for data the ages of physicians and surgeons of eminence, it would seem that the anxieties of medical life, the loss of rest, exposure to infectious or contagious disease, were not unfavorable to longevity. If a medical man commences medical life with a fairly balanced constitution, and with what is of still more or equal importance, a fair balance to his credit at his bankers, the chances are that he will both secure a good practice and live to a good age, provided he follows fairly the general laws of health.

If, on the other hand, his health is exceptionally good, and he is but scantly provided with funds, he will have greater difficulty in acquiring a good *clientele*, whilst the worry and anxiety about pecuniary matters will have a tendency to shorten his life.

It is the worry about ways and means that kills. Casper affirmed that German medical practitioners were shorter-lived. Dubois has ascertained that out of 800 practitioners, 365 attained the age of 70 and upwards. This exceeds the proportion allotted to theologians. Accurate modern statistics are difficult to secure, but we feel certain it will be found the mortality will vary in proportion to the pecuniary position of the individuals.—*The Medical Press and Circular.—Cincinnati Lancet and Clinic.*

